# Selected Cannabinoids, Cannabimimetic Agents and *Artemisia* Combinations as Theoretical Adjunct Strategies Against COVID-19

**DOI:** 10.3390/ph19060869

**Published:** 2026-05-30

**Authors:** Harry Chiririwa

**Affiliations:** Department of Natural Sciences, Vaal University of Technology, Andries Potgieter Blvd, Vanderbijlpark 1900, South Africa; haledenc@vut.ac.za

**Keywords:** COVID-19, cannabinoids, cannabimimetics, *Artemisia*, natural therapeutics, phytochemicals, SARS-CoV-2, antiviral activity, immunomodulation, complementary medicine

## Abstract

COVID-19 has spurred much interest in complementary and alternative agents for therapeutic purposes having antiviral and immunomodulatory effects. In these, natural products and bioactive compounds from plants have been at the center of attention due to their easy access, relatively low risk and long history of use in traditional medicine. This paper reviews in detail and critically assesses the scientific data that presently proposes the use of certain cannabinoids, cannabimimetic compounds and *Artemisia* species in the treatment and prevention of COVID-19. It gives an account of medicinal approaches to cannabinoids like cannabidiol (CBD), Δ9-tetrahydrocannabinol (THC) alongside other minor cannabinoids and synthetic and naturally-occurring cannabimimetics. The paper reports the potential of *Artemisia annua* and other species as treatments, especially focusing on their antiviral, anti-regulatory, anti-inflammatory and immunomodulating properties. It highlights the molecular interactions with SARS-CoV-2 targets as well as cytokine regulation and modulation of oxidative stress pathways, with special emphasis on these areas. The paper raises multiple issues like preclinical and clinical studies, safety aspects, regulatory hurdles and drawbacks related to the use of these natural compounds. After analyzing all the available data, the article entertains the idea of a cannabinoid–*Artemisia* combination as a supportive or adjunct therapy in COVID-19 treatment. It also points out that the clinical trials are insufficient concerning the establishment of effectiveness, determination of the appropriate dosage and assurance of the long-term safety of the treatment.

## 1. Introduction

### 1.1. Background to COVID-19 and Natural Therapeutics

Coronavirus Disease 2019 (COVID-19) is a new disease caused by a virus (SARS-CoV-2) that has seriously challenged health systems, economies and ways of life all over the world [1]. Ever since it was first identified late 2019, COVID-19 has led to millions of infections and deaths globally. It has become extremely important to develop preventive and therapeutic measures as soon as possible [2]. Even though vaccines and antiviral medications greatly help in reducing the severity of the disease and the number of deaths, the emergence of new variants of the virus and the disparity in healthcare access have been the main hindrances to effectively controlling the pandemic [3]. Traditional medical ways to treat COVID-19 mainly revolve around vaccination, antiviral drugs, immune system modulators and supportive care [4]. Nevertheless, such approaches come with their own set of problems such as drug resistance, side effects, high costs of treatment and limited availability in low- and middle-income countries, which have led to a search for other alternatives, namely complementary and alternative therapies. Natural products from medicinal plants have attracted both scientific and public attention as possible beneficial therapies.

Plant-based remedies have been widely relied upon to manage infectious diseases across various cultures and many of the underlying compounds used in modern drugs originate from medicinal plants [5]. Natural medicines generally have many effects like antiviral, anti-inflammatory, antioxidant and immune-modulating, which are all instrumental in the COVID-19 disease process. These effects can not only curb the spread of viruses but can also prevent the cytokine storm, the major cause of disease severity and relieve the symptoms [6]. During the COVID-19 pandemic several herbal combinations and plant chemicals were tested for their potential roles in protection and treatment. Flavonoids, terpenoids, alkaloids and polyphenols are some of the plant-derived compounds that have potently inhibited the virus from entering and replicating in cells in in vitro experiments [7]. Along with this, traditional medical practices in Africa, Asia and South America also saw more use of plant-based remedies for immune boosting and lung health [8].

The scientific validation of many natural therapies continues to be limited, although many people are becoming interested in using them. There are challenges such as the fact that plant composition can vary, dosages are not standardized and there are not enough clinical trials, making it difficult for the medical community to accept them as part of a standard treatment [9]. Systematic reviews and evidence-based assessments are needed to identify which ones are good and which ones are just making claims without evidence. This paper reports on investigating the possible role of some cannabinoids and cannabimimetic compounds together with the *Artemisia* genus as alternative and complementary therapeutic agents at the time of COVID-19. Through in-depth analysis of previous studies, this paper aims at informing the ongoing debate about the advantages, disadvantages and possibilities of natural treatment methods in caring for viral pandemics.

Combination-based therapeutic strategies have attracted much interest in antiviral research as they offer several potential pharmacological advantages over single-agent interventions [10]. By simultaneously targeting multiple viral and host pathways, the combination therapies enhance overall therapeutic efficacy while decreasing reliance on a single mechanism of action. These multi-target approaches help mitigate the emergence of viral resistance, a well-recognized challenge in antiviral drug development. Synergistic or additive interactions between bioactive compounds permit the use of decreased therapeutic doses thereby improving tolerability and reducing toxicity. Cannabinoids have been investigated for their immunomodulatory, anti-inflammatory and symptom-relieving properties while *Artemisia*-derived compounds have shown antiviral, antioxidant and host-response modulatory activities in various experimental models. The theoretical combination of these agents guarantees research as adjunct strategies in COVID-19, particularly in targeting several pathological processes including viral replication, oxidative stress, inflammation and dysregulated immune responses. However, such approaches remain largely theoretical and require rigorous experimental and clinical validation.

### 1.2. Rationale for Plant-Based and Cannabinoid Therapies

The ongoing search for effective therapies for COVID-19 has shown that it is important to look at different pharmacological methods including those taken from nature. Medicines from plants and compounds related to cannabinoids have been identified as potential candidates owing to their multiple biological activities, extensive traditional use and scientific confirmation [11]. Their possible usefulness in handling viral infections depends on their capability to modulate several disease pathways at the same time. A major reason for considering therapies based on plants is their abundance of bioactive phytochemicals such as flavonoids, terpenes, phenolics, alkaloids and glycosides [12]. Besides being antiviral, anti-inflammatory, antioxidant and immunomodulatory agents, these compounds also possess other beneficial attributes. Concerning COVID-19, these multifunctional effects are very important because the disease involves not only viruses but also immune system disruption, oxidative stress and inflammatory damage to tissues. Compared to single-target synthetic drugs, plant-derived compounds may have combined effects that result in better therapeutic effects [13].

**Figure 1:**
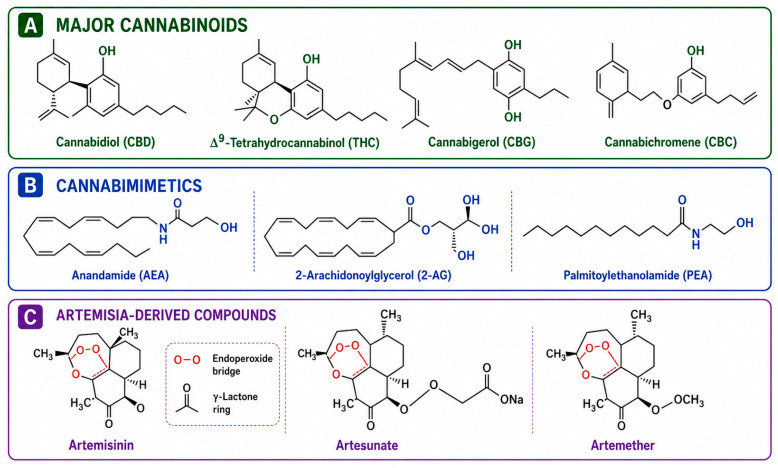
Chemical structures of selected cannabinoids, cannabimimetics and *Artemisia*-derived compounds with potential therapeutic relevance against COVID-19. Adapted from [18].

Cannabinoids and cannabimimetic compounds are a special set of bioactive molecules that are becoming increasingly important in medical research (Figure 1). Natural cannabinoids like cannabidiol (CBD), Δ9-tetrahydrocannabinol (THC) and less abundant cannabinoids influence the endocannabinoid system that among its other functions regulates immune responses, inflammation pain and overall balance [14]. Changing the activity of this system affects cytokine production, leukocyte functions and inflammatory pathways, hence leading to the possibility of controlling the hyperinflammatory responses characteristic of severe COVID-19 [15]. Besides the cannabimimetic compounds, synthetic analogues or natural molecules having cannabinoid activity provide better pharmacological stability and selectivity. Such compounds can imitate or modulate endocannabinoid signaling without triggering strong psychoactive effects, thus they are very attractive potential therapeutics [16]. Their capacity to maintain immune equilibrium and to alleviate tissue inflammation provides the rationale for their adjunct treatment in viral infections. Plant and cannabinoid therapies also receive support from practical and socio-economic standpoints. Medicinal plants are accessible worldwide, particularly in resource-limited areas where they often serve as less expensive substitutes to brand-name drugs. Their inclusion into health care systems may lead to greater availability of auxiliary treatments in public health crises [17]. After proper standardization and administration, several plant-derived products exhibit a good safety profile.

Recent computational, laboratory and animal-model studies have added to the evidence base supporting the investigation of these treatments against the novel coronavirus. Molecular docking studies have indicated that certain plant-derived compounds and cannabinoids can bind to viral proteins such as the main protease (Mpro), spike protein and RNA-dependent RNA polymerase [19]. Biological assays have demonstrated that these substances can prevent viral entry and replication and modulate immunity of the host to some extent. The translation of plant-derived and cannabinoid therapies into clinical practice must be handled with caution as factors like dose standardization, bioavailability, drug interactions and safety upon long-term use remain as major hurdles that need to be overcome [20]. Thorough scientific confirmation and well-designed clinical trials are required before these treatments can be widely used. The justification for investigating plant-based and cannabinoid therapies in COVID-19 management lies in their wide spectrum of pharmacological activities, immunoregulatory capacity, availability and increasing scientific data [21]. These features provide ample reasons for the continued investigation of their potential as complementary and supportive therapeutic interventions in the control of viral infections.

### 1.3. Scope and Objectives of the Review

This paper aims to describe the potential role of some cannabinoids, cannabimimetic compounds and *Artemisia* species in the prevention, management and supportive treatment of COVID-19. It explores the scientific literature to give a comprehensive and critical synthesis of current knowledge about their pharmacological properties, mechanisms of action, therapeutic importance and limitations in the context of SARS-CoV-2 infection. This paper reviews preclinical, clinical and computational studies that have examined the antiviral, anti-inflammatory, antioxidant and immunomodulatory properties of these natural compounds. It mainly focuses on studies that investigate molecular binding to viral targets, changing host immune responses and possible synergistic effects of combination therapies. It also considers the conventional and ethnopharmacological uses of cannabis-derived compounds and *Artemisia* species in the management of respiratory and infectious diseases. This paper attempts to examine major areas like safety toxicity dosage standardization, regulatory frameworks and ethical considerations of medicinal uses of plant-based and cannabinoid products. It also discusses problems in the quality control of products, differences in the chemical composition of plants and possible drug–drug interactions especially for patients being treated with standard COVID-19 regimens.

The primary objectives of this paper follow:

1.3.1. To study the scientific investigations about the antiviral and immunomodulatory effects of specific cannabinoids, cannabimimetics and *Artemisia* compounds against SARS-CoV-2.

1.3.2. To investigate the molecular and cellular mechanisms behind the impact of these compounds on virus entry and disease development.

1.3.3. To identify the practical value, potential risks and inefficiencies of using these natural products as therapeutics.

1.3.4. To locate the missing elements and weak points of the experimental designs of present studies.

1.3.5. To suggest future dissertation topics and clinical research that would prove or disprove the effectiveness of combination therapies.

This paper intends to promote the evidence-based comprehension of herbal therapies for COVID-19 and assist the decision-making of researchers, clinicians and policymakers who wish to consider these methods as a part of the healthcare system.

## 2. Overview of COVID-19 Pathophysiology

### 2.1. SARS-CoV-2 Structure and Replication

Severe Acute Respiratory Syndrome Coronavirus 2 (SARS-CoV-2), which belongs to the *Coronaviridae* family and the genus *Betacoronavirus*, is an enveloped positive-sense single-stranded RNA virus [22]. As the causative agent of Coronavirus Disease 2019 (COVID-19), it presents a wide range of symptoms starting from asymptomatic infection to severe respiratory failure and multi-organ dysfunction. In-depth knowledge of the structural and replicative characteristics of SARS-CoV-2 not only aids in the unveiling of new therapeutic targets but also establishes the way for understanding how natural compounds might manifest antiviral properties through various mechanisms [23].

### 2.2. Structural Organization of SARS-CoV-2

The particles of SARS-CoV-2 can be either round or irregular in shape, and their size is mostly between 60 and 140 nm. The virus is marked by very recognizable spike projections on its outer surface that make a coronavirus resemble a crown under the microscope. The virus’ genetic material is protected within a lipid envelope that the virus acquires from the host cell membrane [24].

The viral structure consists of four major structural proteins:

2.2.1. Spike (S) protein—A glycoprotein responsible for viral attachment and entry into host cells. The S protein binds to the angiotensin-converting enzyme 2 (ACE2) receptor on host cells and facilitates membrane fusion following activation by host proteases such as TMPRSS2.

2.2.2. Envelope (E) protein—A small membrane protein involved in viral assembly, release, and pathogenicity.

2.2.3. Membrane (M) protein—The most abundant structural protein, which maintains viral shape and coordinates virion assembly.

2.2.4. Nucleocapsid (N) protein—Binds to viral RNA to form a ribonucleoprotein complex and plays a role in genome packaging and replication.

In addition to these structural proteins, SARS-CoV-2 encodes several non-structural and accessory proteins that regulate viral replication, immune evasion and host–virus interactions [25].

### 2.3. Viral Entry and Attachment

The first step of an infection is the viral spike protein attaching to the ACE2 receptor, which is abundantly present in the cells lining the respiratory tract, and also in the cells of blood vessels and various parts of the body including the heart, kidneys and digestive system. After the virus binds to the receptor the host enzymes cleave the S protein at certain locations. These cuts cause the protein to change shape [26]. As a result the virus’s envelope can merge with the membrane of the host’s cell. Once this merging has taken place the viral RNA is released into the host cell’s cytoplasm where it undergoes replication and translation. Since the efficiency with which a virus enters a cell largely influences both how infectious the virus is and the severity of the resulting illness, the process of viral entry represents a key target for developing antiviral therapies [27].

### 2.4. Replication and Transcription Cycle

After entering the host cell the viral RNA genome immediately acts as a messenger RNA. Replication begins with translating the viral genome into two huge polyproteins, pp1a and pp1ab. These polyproteins are broken down by viral proteases, such as the main protease (Mpro) and papain-like protease (PLpro), into non-structural proteins (nsps) that are necessary for the virus to replicate [28]. These non-structural proteins come together to create the replication transcription complex (RTC), which is linked with modified intracellular membranes that originate from the endoplasmic reticulum. The RTC is responsible for creating negative-sense RNA intermediates that are then used as templates to make new genomic RNA and subgenomic mRNAs. Subgenomic mRNAs are used to make structural and accessory proteins that are moved to the endoplasmic reticulum Golgi intermediate compartment (ERGIC) [29]. The virus is assembled in this compartment where the freshly made genomic RNA first binds with nucleocapsid proteins and then enclosed by a membrane containing S, M and E proteins [30].

### 2.5. Virion Assembly and Release

Once assembled, mature virions are enclosed in vesicles and moved to the cell surface using the secretory pathway. The virions leave the cell by exocytosis, which helps them to spread and infect nearby cells, furthering the infection in the host. A fast replication process of this virus results in significantly high viral loads mainly during the initial stages of infection. The ongoing synthesis and secretion of viral particles cause serious cellular injuries, breakdown of epithelial barriers and stimulation of innate immune defenses. Such processes are crucial in triggering the series of inflammatory events that lead to the development of COVID-19 [31].

### 2.6. Implications for Therapeutic Targeting

Many components of SARS-CoV-2 that are structural and for replication serve as a basis for various antiviral interventions; these include the interaction spike ACE2, viral proteases, RNA-dependent RNA polymerase (RdRp) and assembly pathways [32]. Many natural compounds including cannabinoids and phytochemicals from *Artemisia* have been proposed for their potential to interfere with these processes through either the direct binding or modulation of host factors. Knowledge of SARS-CoV-2 structure and its replication not only makes clear the mechanisms of the disease but also helps in the logical development of plant-based and cannabinoid-derived therapeutic strategies [33].

### 2.7. Host Immune Response and Cytokine Storm

The immune response of the host plays an important role in determining the clinical outcome of SARS-CoV-2 infection. When the virus first enters the body, a successful innate immune response is one of the factors that the body uses to determine whether the virus can be cleared [34]. Receptors that recognize pathogens including toll-like receptors (TLRs) become activated, detect the viral RNA and trigger the production of type I interferons and pro-inflammatory cytokines. These early responses are critical for limiting viral replication and initiating adaptive immunity [35]. In severely ill COVID-19 patients this precisely controlled immune response may become disorderly and cause the body to overreact with an inflammatory response that is sometimes referred to as a “cytokine storm“. This hyperinflammatory state is the result of overproduction of cytokines and chemokines including interleukin (IL)-6, IL-1, tumor necrosis factor-alpha (TNF-α), IL-8 and interferon-gamma (IFN-γ) [36]. The uncontrolled and extensive production of these mediators results in the infiltration of immune cells and to a great extent the dysfunction of the endothelium, leakage in the blood vessels and finally damage to several organs especially the lungs in the form of acute respiratory distress syndrome (ARDS) [37].

A cytokine storm is characterized by a vicious cycle of increased inflammation due to pro-inflammatory cytokines overriding the anti-inflammatory cytokines. In normal immune responses regulatory mechanisms such as regulatory T cells and anti-inflammatory cytokines (IL-10) will help to eliminate inflammation [38]. In severe cases of COVID-19, these regulatory systems are either inhibited or insufficient, thus enabling inflammation to continue unabated. This scenario is worsened by a delayed type I interferon response, which results in a lack of early viral control and prolonged immune system overdrive [39]. Overworked monocytes and macrophages are the main factors in the continuation of systemic inflammation. These cells penetrate the lung tissue and secrete many inflammatory messages, causing more damage to the tissue. The activation of endothelium and abnormalities in coagulation result in complications in very sick patients, such as thrombosis and disseminated intravascular coagulation [40].

For devising specific treatment approaches, it is crucial to understand the mechanisms that cause immune dysregulation in the host and the production of the cytokine storm. Immunoregulatory therapies that aim to inhibit the overproduction of cytokines or to bring the immune system back to its normal state, for example, IL-6 receptor blockers and corticosteroids, have shown benefits at the clinical level in severe cases. The exact moment of intervention and proper patient selection are very important to prevent weakening the protective antiviral immunity [41]. The cytokine storm is a major pathological event in severe COVID-19 that progresses from the viral infection to systemic inflammatory damage and it is the focus of therapeutic efforts.

### 2.8. Targets for Therapeutic Intervention

Approaches to treat COVID-19 and other virus-caused diseases are mainly based on understanding of the viral life cycle and the immune response that is not properly functioning in the host [42]. Discovering molecular and cellular targets both in viral and host pathways has allowed the creation and re-use of drugs that aim to reduce viral replication, limit tissue damage and improve clinical outcomes [43]. A major therapeutic focus is the process through which the virus enters the host cells. The mechanism permitting the virus to initiate infection is the viral spike (S) protein attaching to the angiotensin-converting enzyme 2 (ACE2) receptor, which is prepared for entry by the transmembrane protease serine 2 (TMPRSS2) [44]. Treatments that prevent spike-ACE2 interaction or that stop TMPRSS2 activity can effectively block viral entry and multiplication. Neutralizing monoclonal antibodies and entry inhibitors are the examples of drugs that were created with the aim of disrupting this pivotal step. Another crucial target in this regard is the viral replication machinery [45]. After gaining access to the host cell, SARS-CoV-2 depends on viral enzymes such as RNA-dependent RNA polymerase (RdRp) and proteases, including the main protease (Mpro/3CLpro) and papain-like protease (PLpro), to replicate and process viral proteins. Enzyme inhibitors are capable of drastically decreasing the production of viruses. For instance, nucleoside analogues work by causing premature termination of the synthesis of viral RNA, thus limiting the infection spread [46].

Host-directed therapies have been gaining traction as key intervention strategies. Severe disease is often the result of an overzealous immune response, which is why inflammatory pathways are targeted for a complementary approach. Major elements are cytokines, such as interleukin-6 (IL-6), interleukin-1 (IL-1) and tumor necrosis factor-alpha (TNF-α), alongside their corresponding signaling pathways (JAK-STAT signaling). Manipulation of these pathways can aid in the prevention or decrease in cytokine storm-related tissue damage [47]. Coagulation pathways make an appealing target for therapy given that thrombotic complications are very common in severe COVID-19. Drugs that influence platelet activation and coagulation systems are implemented for lowering the risks of microthrombosis and subsequent organ failure [48]. New studies point out the possibility of using host metabolic and oxidative stress pathways as a target to combat viral infection, which triggers major cellular stress responses [49]. Besides antioxidants, immunomodulatory phytochemicals or plant-based compounds are being investigated for their potential to provide a redox balance and control immune activation [50]. A suitable therapeutic intervention needs a multi-target approach that combines antiviral tactics with host immune modulation. Such dual attention is indispensable for not only halting the proliferation of virus but also lessening the harmful effects of immune dysregulation in the case of severe disease.

## 3. Cannabinoids and Cannabimimetic Compounds

Cannabinoids and cannabimimetic compounds form a diverse group of biologically active chemicals that target the endocannabinoid system (ECS) which is a multifaceted regulatory network playing a role in immune modulation inflammation pain perception and cellular homeostasis [51]. The potential therapeutic applications of these compounds in inflammatory and infectious diseases, including COVID-19 have greatly increased global interest in their use.

### 3.1. Overview of Cannabinoids and the Endocannabinoid System

Cannabinoids are divided into three main categories: phytocannabinoids obtained from *Cannabis sativa*; endogenous cannabinoids, also called endocannabinoids, which are produced by the human body; and synthetic cannabinoids that are made to resemble or influence cannabinoid receptor activity [52]. The primary cannabinoid receptors CB1 and CB2 are extensively distributed in the central nervous system and peripheral immune tissues, respectively. CB1 receptors are primarily linked to brain functions whereas CB2 receptors are mostly found on immune cells such as macrophages, B cells and T cells [53]. The role of CB2 receptor activation in immunomodulation is very significant as it helps in controlling excessive inflammatory reactions without producing strong psychoactive effects. CB2 is considered a major target in the treatment of inflammatory diseases [54].

### 3.2. Cannabinoids and Immune Modulation

Cannabinoids have a capacity to influence the immune system by controlling cytokine production, the movement of immune cells and the pathways of inflammatory signaling [55]. Research shows that certain cannabinoids can decrease the production of cytokines involved in inflammation such as IL-6, TNF-α and IL-1 while at the same time increasing the production of anti-inflammatory cytokines like IL-10. This alteration in the cytokine profile may play a role in lessening the impact of hyperinflammatory states like cytokine storm syndrome in very ill COVID-19 patients. Cannabinoids influence major signaling pathways that are responsible for immune system activation such as the NF-κB and JAK-STAT pathways. Through the alteration of these pathways, cannabinoids lessen the over-activation of the immune system and the resulting tissue injuries and yet remain capable in executing antiviral immune defense mechanisms [56].

### 3.3. Cannabimimetic Compounds

Cannabimimetic compounds are synthetic or naturally occurring molecules that imitate the effects of cannabinoids by targeting cannabinoid receptors or related signaling systems. These compounds attract much interest because unlike classical cannabinoids they can be modified chemically for higher potency, selectivity and fewer psychoactive effects [57]. Synthetic cannabinoids have been designed to selectively bind to CB2 receptors, which leads to an immunomodulatory response while keeping side effects to the central nervous system at a minimum [58]. Some non-cannabinoid plant-based compounds have also been found to have cannabimimetic properties via their influence on endocannabinoid metabolism or receptor signaling in an indirect way [59].

### 3.4. Therapeutic Potential in Viral and Inflammatory Diseases

Immunomodulatory features of cannabinoids and cannabimimetic substances imply there could be a therapeutic role for them in viral infections with an excessive inflammatory component. In COVID-19 such substances might mitigate cytokine storms, decrease inflammation of the lungs and prevent tissue damage. Through their antioxidative and anti-cell death mechanisms they might aid in safeguarding cells experiencing oxidative stresses due to viruses [60]. Preliminary laboratory results point to the potential of these compounds, but patient data is not available and more rigorous controlled trials must be completed to produce data on effectiveness safety dosage and long-term effects.

### 3.5. Limitations and Research Considerations

There are several difficulties that need to be resolved along with the cannabis therapeutic potential. These challenges include variability in chemical compound profiles of cannabis, divergences in receptor selectivity, possible drug–drug interactions and regulatory controls on cannabis-based products. The immunosuppressive effects of cannabinoids might interfere with antiviral immune defense if not properly regulated [61]. Cannabinoids and cannabimimetic compounds are a class of immunomodulatory agents that are very promising with respect to inflammatory and infectious diseases [62]. Since their capability to control cytokine production and immune signaling pathways is effective, they can be considered as adjunctive therapy for diseases like COVID-19 [63]. More mechanistic investigations and properly designed clinical trials are indispensable before their habitual therapeutic use can be advised.

## 4. *Artemisia* Species in Antiviral Therapy

Through time, various species of the *Artemisia* genus have been utilized as a part of traditional medicine to combat ailments such as fever, infections and inflammation [64]. *Artemisia annua* (sweet wormwood) and *Artemisia afra* (African wormwood) have been the subjects of numerous studies as they have very potent phytochemicals and a wide range of effects on microbes and viruses have been reported. As the world is facing a threat of new viral diseases, there has been a surge in interest in these plants, including their potential role in the fight against COVID-19 [65].

### 4.1. Phytochemical Constituents and Bioactive Compounds

The medicinal properties of *Artemisia* mainly depend on their wide variety of secondary metabolites like sesquiterpene lactones, flavonoids, terpenoids and essential oils. The most famous is *artemisinin*, a sesquiterpene lactone endoperoxide derived from *A. annua*, which is a powerful antimalarial drug [66]. Plant flavonoids including quercetin, luteolin and kaempferol largely participate in the antioxidant and anti-inflammatory actions of the herb. These chemicals together can manifest various biological effects such as scavenging free radicals, inhibiting enzymes and altering immune responses, which may relate to viral infections and their complications in inflammation [67].

### 4.2. Antiviral Mechanisms of Action

The antiviral effect of *Artemisia* is thought to be a combination of several mechanisms. Both in silico and in vitro experiments have shown that *artemisinin* and the derivatives might impact virus replication through the inhibition of the main viral enzymes and the interruption in protein synthesis [68]. The flavonoids in *Artemisia* plants have the potential to block viral penetration and replication by the interaction with host and viral proteins. A significant mechanism is the influence on host immune responses [69]. Samples of *Artemisia* have shown the capability in regulating cytokine production. They decrease the production levels of pro-inflammatory molecules such as IL-6 and TNF- α and at the same time increase the antioxidant defense system [70]. This effect of modulating the immune system is very important, especially where there is an overproduction of inflammation such as the cytokine storm associated with COVID-19.

### 4.3. Relevance to COVID-19 Research

During the COVID-19 outbreak *Artemisia* plants became the focus of studies as possible supportive treatment in view of their antiviral and anti-inflammatory activities, which are substantiated by many studies [71]. It has been hypothesized through computer-aided docking that the molecules of *A. annua* may be capable of interacting with the proteins of SARS-CoV-2, such as the main protease (Mpro) and the spike receptor-binding domain, thereby exerting an inhibitory effect [72]. However, based on these promising results there is only limited and inconsistent clinical data. Some initial reports and small-scale studies have pointed to possible benefits but there is a lack of strong trials. Hence the effectiveness of *Artemisia*-based therapies for COVID-19 in clinical settings has not yet been proven [73].

### 4.4. Safety, Toxicology, and Standardization Challenges

Since *Artemisia* plants have been traditionally used without major harmful effects, they are generally considered safe. However, there are considerable issues behind changes in the ways of new preparations, dosage and phytochemical composition [74]. The level of the active substances depends largely on conditions of the environment, methods of obtaining and parts of the plant used. There is some worry about the possibility of harmful effects when taken at high doses or for a long time, particularly with *artemisinin* derivatives. The combination with conventional antiviral or antimalarial drugs may change the results of the treatment so an accurate assessment of safety is required.

## 5. Mechanisms of Action Against SARS-CoV-2

**Figure 2:**
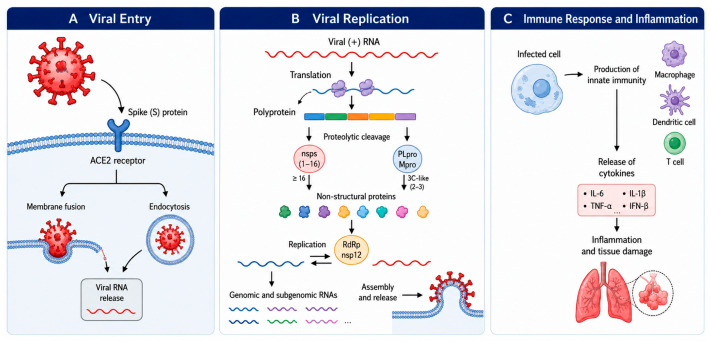
Mechanism of action of cannabinoids, cannabimimetics and *artemisia* compounds against SARS-CoV-2. Adapted from [77,78].

The antiviral mechanisms behind cannabinoids, cannabimimetic compounds and *Artemisia* species against SARS-CoV-2 (Figure 2) have been hypothesized to result from a mixture of direct antiviral activities targeting the viral life cycle and indirect regulation of host inflammatory and immune responses [75]. Such multi-targeted actions are very apt for a situation like COVID-19, where it is not only the SARS-CoV-2 viral replication but also the severely dysregulated host immunity that are responsible for the worsened clinical outcomes [76].

### 5.1. Direct Inhibition of Viral Entry

Infection by SARS-CoV-2 is initiated at an early stage by the spike (S) protein of the virus, which latches onto the angiotensin-converting enzyme 2 (ACE2) receptor on the host cell surface, and this is followed by the spike protein being cleaved and activated by the host protease(s) such as TMPRSS2 [79]. Bioactive ingredients of cannabinoids and *Artemisia* species have been implied based on in silico and in-vitro models to disrupt this phenomenon. Some plant compounds can attach to the part of the spike protein that binds to the receptor, resulting in a lowered binding capacity to ACE2, while others can alter the expression of ACE2 or block the activity of the protease TMPRSS2. Such dual mode of inhibitions can potentially decrease the virus sticking and entering the cell, therefore restricting the initial infection and viral spread [80].

### 5.2. Disruption of Viral Replication and Protein Processing

After entering the host cell, the coronavirus depends on the help of its RNA-dependent RNA polymerase (RdRp) and the proteolytic enzymes such as the main protease (Mpro/3CLpro) and the papain-like protease (PLpro) to synthesize mature viral cells [81]. In silico studies have found that several bioactive elements deriving from *Artemisia*, especially *artemisinin* and flavonoid derivatives and a few cannabinoids from the selected group, show promising inhibition exposure towards the viral enzymes studied. These substances may lessen the production of viral RNA by hindering the activity of RdRp. The blocking of the viral proteases that process the cleavage of polyproteins into functional units may not only lead to the disruption of viral maturation and assembly but also to additional viral elimination [82]. Most of these results are of a computational and preclinical nature meaning that they serve as a framework for the experimental validations to be carried out later.

### 5.3. Modulating the Host Immune Response

The host immune response, particularly the development of hyperinflammation and cytokine storm, is a crucial factor determining the severity of COVID-19. *Cannabis* and *Artemisia* compounds have immunomodulatory capabilities that can help in normalizing the immune system. Such compounds suppress the production of pro-inflammatory cytokines IL-6, IL-1 and TNF, whereas they promote the production of the anti-inflammatory mediator IL-10. This cytokine regulation is tied to a diminished activation of major inflammatory pathways such as NF-κB and JAK-STAT signaling and as a result overactivation of immune response and destruction of tissues might be diminished.

### 5.4. Antioxidant and Cytoprotective Effects

One of the consequences of SARS-CoV-2 infection is oxidative stress due to the release of large amounts of reactive oxygen species (ROS) that damage lung tissue [83]. Flavonoids and terpenoids found in *Artemisia* species and certain cannabinoids are potent antioxidants. The three-dimensional conformation of cannabinoids greatly influences their biological and antioxidant activities by affecting receptor binding, hydrogen bonding and hydrophobic interactions. The arrangement of functional groups, particularly phenolic hydroxyls and aromatic rings (Table 1), determines their ability to modulate oxidative stress, inflammation and cannabinoid receptor interactions relevant to SARS-CoV-2-associated inflammatory pathways [84].

These molecules can directly neutralize free radicals as well as stimulate production of the body’s own antioxidants like glutathione and superoxide dismutase (SOD). By lowering oxidative stress, they could not only prevent endothelial and epithelial cell damage but also improve tissue response to infection [88].

### 5.5. Anti-Thrombotic and Endothelial Protective Effects

Severe COVID-19 is most often associated with endothelial injury and dysregulated coagulation resulting in microthrombosis and multiple organ failure [89]. Certain cannabinoids are reported to influence platelet function and vascular tone while both *Artemisia* compounds and their metabolites can increase endothelial cell function by the combination of anti-inflammatory and antioxidant mechanisms. Although the exact roles of these mechanisms in SARS-CoV-2 infection have yet to be uncovered, it can be inferred that they may help mitigate the thrombo-inflammatory complications of the disease [90].

### 5.6. Integrated Multi-Target Action

The mechanisms proposed for cannabinoids, cannabimimetic compounds and Artemisia species against SARS-CoV-2 extend beyond a single pathway but rather consist of a complex set of interactions that, among other factors, inhibit viral entry and replication, modulate immune signaling and oxidative stress and maintain vascular integrity. A multi-target profile of this kind is especially beneficial in a complicated disease like COVID-19 where both the virus and the host contribute to the development of the disease [91].

The antiviral property of these natural and synthetic agents is due to antiviral interactions that directly influence viral activity and host-directed effects. While preclinical data are quite encouraging, more experimental studies and clinical trials are needed to validate such mechanisms and to evaluate their therapeutic potential in COVID-19 treatment.

## 6. Future Perspectives on Synergistic Combination Therapies

It has been suggested that the mixture of cannabinoids, cannabimimetics and compounds derived from *Artemisia* could be used to increase therapeutic effects in COVID-19 in a complementary and possibly synergistic way. Instead of a single mechanism of action, these agents are involved in numerous biological processes of SARS-CoV-2 such as viral entry, immune dysregulation and inflammation. Phytocannabinoids, including cannabidiol (CBD) and -tetrahydrocannabinol (THC), show immunomodulation by mainly influencing cytokine production and immune cell signaling via their interaction with the endocannabinoid system [92]. This is expected to contribute to mitigating the excessive inflammatory response in severe cases of COVID-19. Cannabimimetics designed to replicate cannabinoid receptor activity may even potentiate the immunoregulatory effects by targeting similar pathways while not necessarily sharing the same chemical structures. *Artemisia annua*-derived substances, mainly *artemisinin* and its derivatives, appear to be antiviral, anti-inflammatory and antioxidant [93]. These substances may act against viral replication and oxidative damage while supporting cannabinoids’ immunomodulatory actions (Table 2).

The combined mechanisms of cannabinoids and compounds from *Artemisia* not only offer the possibility of a multi-target therapy strategy, but also indicate that cannabinoids could mainly regulate immune responses whereas *Artemisia* compounds can carry out antiviral and anti-inflammatory functions. Possible synergy of these compounds may include their multi-biological pathway targeting, cytokine storm reduction, lower dose requirement for higher therapeutic effects, and lesser side effects through complementary actions.

The evidence for synergistic interactions is mostly limited to in vitro experiments, computational modeling and initial experimental data. There is a lack of large-scale clinical trials proving the safety, efficacy and best dosing of combination therapies in this situation. There are other potential risks such as like drug–drug interactions, differences in compound formulation and regulatory issues, which should be thoroughly assessed. While combination therapies involving cannabinoids, cannabimimetics and *Artemisia*-derived compounds present an intriguing and potentially effective approach, they remain investigational [98]. Further interdisciplinary research, including controlled clinical trials and mechanistic studies, is required to substantiate their role in COVID-19 management.

## 7. Preclinical and Clinical Evidence

A growing body of research has explored the potential of cannabinoids, cannabimimetics and *Artemisia*-derived compounds in the context of COVID-19. However, the strength of evidence varies considerably with most findings derived from preclinical investigations and only limited support from clinical studies [99].

### 7.1. Preclinical Evidence

Preclinical studies including in vitro, in vivo and computational modeling provide initial insights into the biological activity of these compounds. Cannabinoids such as cannabidiol (CBD) and Δ9-tetrahydrocannabinol (THC) have demonstrated anti-inflammatory and immunomodulatory effects through interactions with the endocannabinoid system. These effects include the downregulation of pro-inflammatory cytokines and modulation of immune cell responses, which may be relevant in mitigating the cytokine storm associated with severe COVID-19 [100].

Some studies also suggest that cannabinoids may influence viral entry mechanisms, including modulation of receptor expression, although these findings remain preliminary. Cannabimimetics have shown similar immunoregulatory effects in experimental models, further supporting their potential role in managing inflammatory responses.

Compounds derived from *Artemisia annua*, particularly *artemisinin* and its derivatives, have demonstrated antiviral activity against a range of viruses in laboratory settings. In the context of SARS-CoV-2, these compounds have been reported to inhibit viral replication and reduce oxidative stress. Additionally, their anti-inflammatory properties may complement immune-modulating therapies.

Despite these promising findings, preclinical studies often use controlled conditions that may not accurately reflect human physiology. Variability in experimental design, compound purity and dosing further complicates the interpretation of results.

### 7.2. Clinical Evidence

Clinical evidence supporting the use of these compounds in COVID-19 treatment remains limited and inconclusive. A small number of observational studies and early-phase clinical trials have investigated cannabinoids, primarily CBD, for their potential to reduce inflammation and improve clinical outcomes. While some results suggest symptomatic improvement, these studies are often limited by small sample sizes, lack of control groups, and short follow-up periods [101].

For *Artemisia*-derived compounds including *artemisinin*-based therapies, a few clinical investigations have explored their potential antiviral effects in COVID-19 patients. Some studies report reductions in symptom severity and recovery time; however, methodological limitations such as non-randomized designs and inconsistent dosing limit the reliability of these findings.

There is currently no strong clinical evidence supporting the routine use of cannabinoids, cannabimimetics or *Artemisia*-based therapies as standard treatments for COVID-19. Data on combination therapies remain scarce with no well-designed randomized controlled trials confirming synergistic effects in clinical settings [102].

### 7.3. Key Limitations and Research Gaps

The existing body of evidence is constrained by several important limitations:

- Predominance of preclinical and exploratory studies;
- Lack of large-scale, randomized controlled clinical trials;
- Variability in compound formulations and dosages;
- Limited data on long-term safety and drug interactions;
- Insufficient evidence on combination therapy efficacy.

While preclinical studies suggest that cannabinoids, cannabimimetics and *Artemisia*-derived compounds possess antiviral, anti-inflammatory and immunomodulatory properties, clinical validation remains inadequate. These compounds should therefore be regarded as investigational therapeutic agents, and further rigorous clinical research is required to establish their safety, efficacy and role in COVID-19 management [103].

## 8. Safety, Toxicology, and Dosage Considerations

The use of cannabinoids, cannabimimetics and *Artemisia*-derived compounds as investigational agents against COVID-19 requires careful evaluation of safety, toxicological profiles and dosing strategies. Although these compounds are often perceived as “natural” and therefore safe, their pharmacological activity can lead to significant adverse effects particularly when used inappropriately or without clinical oversight [104].

### 8.1. Safety of Cannabinoids

Cannabinoids such as cannabidiol (CBD) and Δ9-tetrahydrocannabinol (THC) interact with the endocannabinoid system, influencing neurological, cardiovascular and immune functions. CBD is well tolerated; however, reported side effects include fatigue, gastrointestinal disturbances and changes in appetite [105]. THC, in contrast, has psychoactive effects and may cause dizziness, anxiety, cognitive impairment and in some cases dependency [106].

Drug–drug interactions are a critical concern as cannabinoids may inhibit cytochrome P450 enzymes altering the metabolism of commonly used medications such as antivirals, anticoagulants and corticosteroids. This is particularly relevant in COVID-19 patients receiving multiple therapies [107].

### 8.2. Toxicological Considerations of Cannabimimetics

Cannabimimetics that mimic cannabinoid receptor activity may present similar or enhanced pharmacological effects. Synthetic variants have been associated with unpredictable toxicity, cardiovascular complications, neurotoxicity and acute adverse reactions [108]. The lack of standardized formulations and limited toxicological data further complicate their safe application.

### 8.3. Safety of Artemisia-Derived Compounds

Compounds derived from *Artemisia annua* such as *artemisinin* are generally considered safe at therapeutic doses used in antimalarial treatments. However, potential side effects include gastrointestinal discomfort, mild hepatotoxicity and allergic reactions. Prolonged or high-dose use may increase the risk of toxicity particularly in vulnerable populations such as pregnant women and individuals with liver impairment [109].

Variability in plant extracts due to differences in cultivation, extraction methods and compound concentrations can lead to inconsistent safety profiles.

### 8.4. Dosage Challenges

One of the major limitations in the application of these compounds is the lack of standardized dosing guidelines for COVID-19 treatment [110]. Dosage determination is complicated by the following factors:

- Variability in compound purity and formulation;
- Differences in bioavailability and pharmacokinetics;
- Limited clinical trial data to establish therapeutic ranges;
- Potential interactions in combination therapies.

As a result, most dosing regimens remain extrapolated from other therapeutic uses or preclinical studies, which may not be directly applicable to COVID-19 patients.

## 9. Regulatory Considerations

The regulatory status of cannabinoids varies significantly across regions, thus affecting their accessibility and clinical use [111]. THC-containing products are subject to strict control in many jurisdictions due to their psychoactive properties. Similarly, the use of *Artemisia*-based remedies outside approved pharmaceutical formulations raises concerns regarding quality control and safety [112].

While cannabinoids, cannabimimetics and *Artemisia*-derived compounds exhibit promising biological activities, their safety and toxicological profiles require careful consideration. The absence of standardized dosing limited clinical validation and potential for adverse effects highlight the need for rigorous safety assessments. These compounds should therefore be used cautiously and remain within the scope of investigational research until sufficient clinical evidence supports their safe and effective application [113].

### 9.1. Regulatory and Ethical Considerations

The investigation of cannabinoids, cannabimimetics and *Artemisia*-derived compounds as investigational agents against COVID-19 raises important regulatory and ethical issues [114]. These considerations are critical to ensure patient safety, scientific integrity and responsible translation of research findings into clinical practice.

### 9.2. Regulatory Frameworks

The legal status of cannabinoid-based products varies widely across jurisdictions. Compounds containing Δ9-tetrahydrocannabinol (THC) are often subject to strict control due to their psychoactive effects while cannabidiol (CBD) may be more accessible but still regulated depending on its intended use (medical vs. recreational) [115]. Regulatory authorities such as the U.S. Food and Drug Administration and the European Medicines Agency require rigorous evidence of safety, efficacy and quality before approving therapeutic agents [116].

Similarly *Artemisia*-derived products must comply with pharmaceutical standards when marketed as medicines. However, the widespread availability of herbal preparations outside regulated frameworks raises concerns about product quality, consistency and labeling accuracy.

## 10. Ethical Limitations and Future Research Considerations

### Ethical and Regulatory Considerations

Investigational therapies need to follow ethical standards like the Declaration of Helsinki. These include obtaining consent from the patient, weighing risks versus benefits, having the ethical committee giving their approval independently and all results should be made known [117]. The use of unproven therapies outside of clinical trials can jeopardize patient safety and give rise to misinformation, especially if compounds are being marketed as treatments without enough scientific backing. The differences in natural products call for the implementation of strict good manufacturing practice (GMP) standards to guarantee quality, consistency and safety. Scientists must share results accountably by not exaggerating the advantages and at the same time guaranteeing that there is equitable access, affordability and fair intellectual property practices if the therapies are proven. These substances should be kept in controlled research environments until there is very strong clinical evidence.

## 11. Key Limitations and Research Gaps

Currently research into cannabinoids, cannabimimetics and compounds derived from *Artemisia* is primarily at the preclinical stage with very few solid clinical trials being conducted [118]. The data available largely results from small and brief studies with no uniformity in the preparations and dosages used, incomplete knowledge of the mechanisms involved and scarcity of information on combination treatments and safety over a long period. There are also issues such as differing regulations and the probability of publication bias. These shortcomings emphasize the importance of more thorough, well-standardized and clinically oriented investigations.

## 12. Future Research Directions

**Figure 3:**
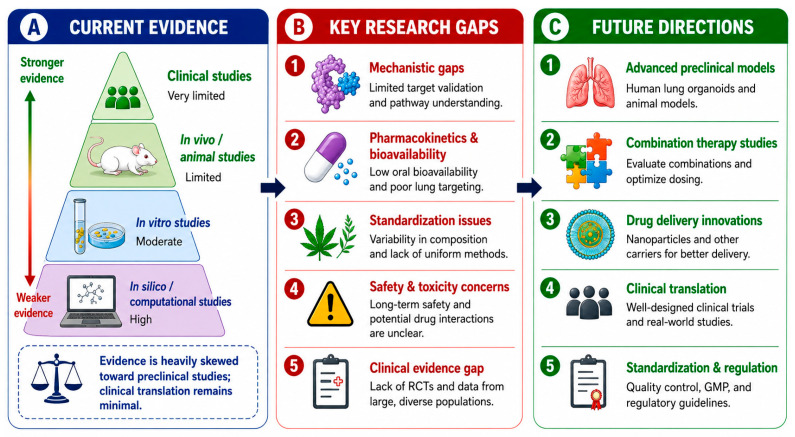
Research gaps and future directions for therapies against COVID-19. Adapted from [119].

Research efforts ought to be directed towards major well-structured randomized controlled trials and the creation of standardized, GMP-grade cannabinoid and *Artemisia* compound formulations. Studies of underlying mechanisms, evaluation of therapeutic combinations, thorough safety investigation, and pharmacokinetic and drug-interaction profiling should be covered in detail. Emphasis should also be put on the development of enhanced drug delivery systems, acquisition of real-world evidence, regulatory harmonization and ethical communication. Most significantly, these agents must be tested as supplemental therapies and not as substitutes for existing COVID-19 treatments (Figure 3).

Research should be directed towards the isolation and identification of individual bioactive compounds, unveiling their pharmacokinetics and assessing the synergistic effects that arise from the confinement of entire plant extracts, among others. Properly constructed clinical trials are indispensable to confirm the effectiveness, safety and suitable dosing schedules. Progress made in the field of nanotechnology and drug delivery methods may increase the bioavailability and stability of Artemisia-derived compounds, thus sharpening their therapeutic potential.

The genus *Artemisia* is a good source of bioactive ingredients demonstrating antiviral, antioxidant and immunomodulatory actions. If preclinical data are indicative of their possible use in the treatment of viral infections with a special focus on the activity against SARS-CoV-2, the time for clinical confirmation is not yet here. Interventions with *Artemisia* extracts should be viewed as complementary forms of treatment rather than definitive antiviral medicine.

## 13. Conclusions

The study of cannabinoids, cannabimimetic molecules and *Artemisia*-based combination therapies points to the increased use of plant and synthetic bioactive molecules in treatment of COVID-19 as possible adjuncts. In silico, in vitro and limited in vivo data shows that these compounds exhibit good antiviral, anti-inflammatory and immunomodulatory activities potentially targeted at SARS-CoV-2 infection and respective pathological manifestations. Nevertheless, the existing literature, despite its positivity, is not enough for a definite clinical application. Most investigations are limited by the absence of standardized dosing, paucity of clinical trials, differences in compound purity, and lack of knowledge of pharmacokinetics and long-term safety profiles. Although a combination of synergistic cannabinoid–*Artemisia* is appealing in theory, it still needs thorough experimental work for proving reproducibility and therapeutic relevance. Research follows the path of not only good preclinical experiments but also clinical trials in defining efficacy, safety and mechanism of action. In view of ethical compliance and patient safety, regulatory frameworks need to change, accommodating scientific evaluation of therapies that are non-traditional. Apart from these natural and synthetic compounds that provide a new avenue in antiviral research, their use in the treatment of COVID-19 is still in the research phase and has not been established by evidence for regular use.

## Figures and Tables

**Table 1:** Structure–activity relationships (SAR) of cannabinoids and cannabimimetic compounds in relation to SARS-CoV-2.

Functional Group	SAR Role	SARS-CoV-2 Relevance	Ref
Phenolic –OH	H-bonding, antioxidant	anti-inflammatory, ACE2 modulation	[85]
Alkyl side chain	lipophilicity, membrane entry	cellular uptake	[86]
Carbonyl (C=O)	enzyme binding	protease docking	[86]
Carboxyl (–COOH)	ionic interactions	spike/ACE2 binding (in silico)	[87]
Aromatic rings	hydrophobic interactions	viral protein docking	[86]
Ether linkage	stability	metabolic persistence	[86]

**Table 2:** Combination and synergistic effects reported within selected cannabinoid and *Artemisia*-derived compound groups.

Combination	Model	Outcome	Evidence of Synergy	Reference
CBD + host antiviral response	Human lung cells	Enhanced interferon signaling and reduced viral replication	Functional synergy with host immune pathways	Nguyen et al., [94]
High-CBD extracts (multi-compound)	Lung fibroblasts	Greater ACE2/TMPRSS2 suppression than single compounds	Suggests phytochemical synergy	Wang et al., [95]
Cannabinoid mixtures (CBD + THC analogs)	In silico + in vitro	Enhanced binding to Mpro	Additive/synergistic docking and antiviral potential	Altyar et al., [18]
*Artemisia annua* extracts (multi-compound)	VeroE6 cells	Higher antiviral activity than isolated compounds	Suggests plant extract synergy	Cao et al., [96]
*Artemisinin* + derivatives (*artesunate*, *artemether*)	In vitro	*Artesunate* shows superior activity vs. *artemisinin* alone	Partial synergy/structure–activity relationship	Cao et al., [94]
Cannabinoids + anti-inflammatory pathways	Preclinical models	Reduction in cytokine storm markers	Functional synergy (anti-inflammatory + antiviral)	Vallée [97]

## Data Availability

No new data were created or analyzed in this study. Data sharing is not applicable to this article.

## Abbreviations

CBD	Cannabidiol
THC	Tetrahydrocannabinol
SARS-CoV-2	Severe acute respiratory syndrome coronavirus 2
Mpro	main protease
PLpro	Papain-like protease
3CLpro	3C-like protease
RdRp	RNA-dependent RNA polymerase
RNA	Ribonucleic acid
mRNA	messenger ribonucleic acid
NSPs	non-structural proteins
RTC	replication transcription complex
ACE2	Angiotensin-converting enzyme 2
TMPRSS2	Transmembrane Protease, Serine 2
TLR	Toll-like receptors
TNF-α	tumor necrosis factor-alpha
IFN-γ	interferon-gamma
IL	Interleukin
ARDS	acute respiratory distress syndrome
NF-κB	Nuclear Factor-kappaB
JAK-STAT	Janus Kinases- Signal Transducers and Activators of Transcription
ECS	endocannabinoid system
CB1	cannabinoid receptor 1
CB2	cannabinoid receptor 2
ROS	reactive oxygen species
